# Supplementary material for: Recent Advances in Chitin Biosynthesis Associated with the Morphology and Secondary Metabolite Synthesis of Filamentous Fungi in Submerged Fermentation
Source: J Fungi (Basel). 2023 Feb 4;9(2):205. doi: 10.3390/jof9020205 (PMC9967639; doi:10.3390/jof9020205)
Supplement: Supplementary file 1 [file jof-09-00205-s001.zip › jof-2176141-supplementary.pdf]

Table S1 The statistical data of the members of chitin synthase family in different species

<table>
<tr><th>Organism</th><th>T-number</th><th>The members of chitin synthase</th><th>Number of genes</th></tr>
<tr><td>Saccharomyces cerevisiae S288c</td><td>T00005</td><td>YBR023C, chs 3
YBR038W, chs 2
YNL192W, chs 1
YLR330W, chs 5
YJL099W, chs 6
YHR142W, no KO assigned | (RefSeq) chs7; chs 7p</td><td>6</td></tr>
<tr><td>Lodderomyces elongisporus NRRLYB-4239</td><td>T01116</td><td>LELG_05384, chs 2
LELG_05013, chs 1
LELG_02210, chs 2
LELG_00298, chs 3
LELG_00300, chs 3</td><td>5</td></tr>
<tr><td>Candida tropicalis MYA-3404</td><td>T01115</td><td>CAALFM_C113110CA, chs 3
CAALFM_C300710WA, chs 8
CAALFM_C702770WA, chs 1
CAALFM_CR09020CA, chs 2</td><td>4</td></tr>
<tr><td>Candida orthopsilosis Co 90-125</td><td>T02488</td><td>CORT_0A01870, chs 3
CORT_0D06430, chs 8
CORT_0G01660, chs 2
CORT_0H01960, chs 1
CORT_0H01970, chs 1</td><td>5</td></tr>
<tr><td>Sugiyamaella lignohabitans CBS 10342</td><td>T05270</td><td>AWJ20_11, chs 6
AWJ20_12, chs 3
AWJ20_13, chs 3
AWJ20_1163, chs 2
AWJ20_1500, chs 2
AWJ20_3769, chs 1
AWJ20_4861, chs 3
AWJ20_4948, chs 3</td><td>8</td></tr>
<tr><td>Artibeus jamaicensis</td><td>T07223</td><td>1119036436, no KO assigned | (RefSeq) CHS3-like</td><td>1</td></tr>
<tr><td>Xenopus laevis (African clawed frog)</td><td>T01010</td><td>108717413, chs 2
108716131, chs 2</td><td>2</td></tr>
<tr><td>Xenopus tropicalis (tropical clawed frog)</td><td>T01011</td><td>105947355, chs 2-like isoform X1</td><td>1</td></tr>
</table>

Danio rerio (zebrafish)	T01004	101886093, *chs* 2 isoform X1	563347, *chs* 2 isoform X1	563138, *chs* 2	322468, *chs* 1							3
Carassius auratus (goldfish)	T07313	113057339 *CHS* 2-like	113061218 *CHS* 1-like	113061224 *CHS* 1-like	113061225 *CHS* 1-like	113061526 *CHS* 1	113061527 *CHS* 1-like	113113123 *CHS* 2-like				7
Ictalurus punctatus (channel catfish)	T04658	108260788, *chs* 1	108260789, *chs* 2	108260790, *chs* 2	108263942, *chs*1	124626088, *chs* 1-like	124627860, *chs* 1-like					6
Pangasianodon hypophthalmus (striped catfish)	T05759	113534702, *chs* 2-like	113534703, *chs* 1-like	113544352, *chs*-like	113544353, *chs* 2-like	113544354, *chs* 2-like						5
Silurus meridionalis	T08004	124381085, *chs* 2-like	124381669, *chs* 1isoform X1,	124381670, *chs* 2-like								3
Neurospora tetrasperma FGSC 2508	T03451	NEUTE1DR AFT75235, *chs* 3	NEUTE1DRAFT 127954, *chs* 1	NEUTE1DR AFT60883, hypothetical protein	NEUTE1D RAFT6088 7, *chs* 6	NEUTE1D RAFT1216 15, *chs* D	NEUTE1DRA FT80490, *chs* A	nte:NEUTE1D RAFT72792, *chs*				7
Pyricularia oryzae 70-15	T01027	MGG_09962, *chs* 4	MGG_06064, *chs* D	MGG_09551, *chs* 3	MGG_1301 3, *chs* 8	MGG_1301 4, *CHS* V	MGG_01802, *chs*1	MGG_04145, *chs* 2				7
Fusarium graminearum	T01038	FGSG_01272 , *chs* 4	FGSG_01949, *chs* D	fgr:FGSG_12 039, *chs* 6	fgr:FGSG_ 01964, hypothetica l protein	fgr:FGSG_ 02483, *chs* 2	fgr:FGSG_101 16, *chs* 1	fgr:FGSG_103 27, *chs* 3	fgr:FGSG_106 19, hypothetical protein	fgr:FGSG _03418, *chs* 1	fgr:FGSG_06550, hypothetical protein	10

Purpureocillium lilacinum	T05029	VFPFJ_00650, *chs* D	VFPFJ_00666, *chs* 6	VFPFJ_00667, *chs* 6	VFPFJ_03324, *chs* D	VFPFJ_04443, *chs* A	VFPFJ_08553, *chs* G	VFPFJ_08866, *chs* A	VFPFJ_11040, chs			8
Scedosporium apiospermum IHEM 14462	T06367	SAPIO_CDS10594, *chs* 1	SAPIO_CDS10760, *chs* 3	SAPIO_CDS3659, *chs* 4	SAPIO_CDS3885, *chs* G	SAPIO_CDS6491, *chs* 1	SAPIO_CDS7076, *chs* 8	SAPIO_CDS7077, *CHS* V	SAPIO_CDS9773, *chs* 2			8
pestalotiopsis fici W106-1	T04924	PFICI_01118, *chs* 1	PFICI_01446, *chs* 4	PFICI_04362, hypothetical protein	PFICI_04363, hypothetical protein	PFICI_05017, *chs* D	PFICI_05238, *chs* 2	PFICI_06085, *chs* 3	PFICI_07201, *chs* 1	PFICI_12982, hypothetical protein	PFICI_13513, *chs* 1	10
Botrytis cinerea B05.10	T01072	BCIN_01g02520, *CHS* IIIb	BCIN_01g03790, *CHS* IV	BCIN_04g03120, *CHS* IIIa	BCIN_07g01300, *CHS* VII	BCIN_09g01210, *CHS* I	BCIN_12g01380, *CHS* II	BCIN_12g05360, *CHS* VI	BCIN_12g05370, *CHS* V			8
Aspergillus fumigatus Af293	T01017	AFUA_4G04180, *chs* B	AFUA_8G05630, *chs* F	AFUA_5G00760, *chs* C	AFUA_2G01870, *chs* A	AFUA_1G12600, *chs* D	AFUA_3G14420, *chs* G	AFUA_2G13430, *chs*	AFUA_2G13440, *chs* E			8
Aspergillus clavatus NRRL 1	T01148	ACLA_022860, *chs* D	ACLA_041870, *chs* G	ACLA_093000, *chs* A	ACLA_054220,*chs* B	ACLA_072240, *chs*, putative	ACLA_072250, *chs* E	ACLA_059050, *chs* F				7
Aspergillus fischeri NRRL 181	T01070	NFIA_005110, *chs*	NFIA_088620, *chs*, putative	NFIA_088630, *chs* E	NFIA_098390, *chs* F	NFIA_029210, *chs* B	NFIA_062840, *chs* G	NFIA_012950, *chs* D	NFIA_034240, *chs* A	NFIA_041420, *chs* C		9
Aspergillus niger CBS 513.88	T01030	ANI_1_316024, chs	ANI_1_2332024, *chs*	ANI_1_1542034, *chs* C	ANI_1_684064, *chs* C	ANI_1_1986074, *chs* D	ANI_1_252084, *chs* D	ANI_1_498084, *chs* B	ANI_1_1214104, *chs* C	ANI_1_120124, *chs* A		9

Aspergillus nidulans FGSC A4	T01016	AN1555.2, *CHS* V (*chs* D)	AN2523.2, *chs* B	AN4367.2, hypothetical protein	AN4566.2, hypothetical protein	AN6317.2, hypothetical protein	AN6318.2, hypothetical protein	AN7032.2, hypothetical protein		7
Neurospora crassa	T01034	NCU09324, *chs* 4	NCU04352, *chs* 5	NCU04350, *chs* 6	NCU05268, *chs* 6;	NCU05239, *chs* A	NCU03611, *chs* 1	NCU04251, *chs* 3		7
Neurospora tetrasperma FGSC 2508	T03451	NEUTE1DRAFT75235， *chs* 3	NEUTE1DRAFT127954， *chs* 1	NEUTE1DRAFT60883， hypothetical protein	NEUTE1DRAFT60887， *chs* 6	NEUTE1DRAFT121615， *chs* D	NEUTE1DRAFT80490， *chs* A	NEUTE1DRAFT72792， *chs*		7
Metarhizium acridum CQMa 102	T03104	MAC_03818, *chs* 1	MAC_07513, *chs* 4	MAC_08014, *CHS* I	MAC_08167, *chs* 2	MAC_08626, *chs* D	MAC_08638, *CHS* III	MAC_08639, *CHS* V		7
Cordyceps militaris CM01	T03108	CCM_00447, *chs* A	CCM_01980, *chs* 1	CCM_02953, *chs* D	CCM_02965, *chs* 6	CCM_02966, *chs*	CCM_06973, *chs* 4	CCM_08096, *chs*	CCM_08511, *chs* 2	8
Colletotrichum fioriniae PJ7	T04796	CFIO01_12076, *chs*	CFIO01_09839, *chs* 3	CFIO01_12746, *CHS* VII	CFIO01_00474, *chs*	CFIO01_00475, *chs*	CFIO01_03762, *chs*	CFIO01_10504, *chs* 4		7
Penicillium digitatum Pd1	T04849	PDIP_79230, *chs* E	PDIP_62350, hypothetical protein	PDIP_46630, *chs* G	PDIP_26990, *chs* D	PDIP_24450, *chs* G	PDIP_15450, *chs* B	PDIP_07640, *chs* A	PDIP_03360, *chs* F	9
Coccidioides immitis RS	T01114	CIMG_05021, *CHS* V	CIMG_05598, *chs* C	CIMG_05647, *chs* G	CIMG_05022, *chs* 5	CIMG_08766, *chs* 4	CIMG_08655, *chs* 2	CIMG_06862, *CHS* VI		8
Zymoseptoria tritici IPO323	T02285	MYCGRDRAFT_51574, hypothetical protein	MYCGRDRAFT_48846, *chs*7	MYCGRDRAFT_100232, *chs* 4 (*CHS* IV)	MYCGRDRAFT_100284, chs2; (*CHS* II)	MYCGRDRAFT_108152, *chs* 5 (*CHS* V)	MYCGRDRAFT_108151, *chs* 6 (*CHS* VI)	MYCGRDRAFT_98274, *chs* 1(*CHS* I)	MYCGRDRAFT_65104, *chs* 3	9

Ustilago maydis 521	T01021	UMAG_10117, *chs* 4	UMAG_10120, *chs* 3	UMAG_10277, *chs* 5	UMAG_10367, *chs* 6	UMAG_03204, *chs* 8	UMAG_04290, *chs* 2	UMAG_10718, *chs* 1	UMAG_05480, *chs* 7			9
**Trichoderma reesei QM6a**	T02991	TRIREDRAFT_55341，*chs*	TRIREDRAFT_58188，*chs*	TRIREDRAFT_12217，*chs*	TRIREDRAFT_71563，*chs*	TRIREDRAFT_67600，*chs*	TRIREDRAFT_51492，*chs*	TRIREDRAFT_112271，*chs*	TRIREDRAFT_124228，*chs*			8
Chaetomium thermophilum var. thermophilum DSM 1495	T03100	CTHT_0014410, no KO assigned	CTHT_0029870, *chs*	CTHT_0062640, *chs*	CTHT_0030060, *chs*	CTHT_0030070, *chs*	CTHT_0039460, hypothetical protein	CTHT_0051660, *chs*	CTHT_0049020, no KO assigned	CTHT_0059700, no KO assigned	CTHT_0059710, *chs*	10
Monascus purpureus HQ1		TQB77221.1, *CHS* V	TQB75461.1, *CHS* III	TQB73913.1, *CHS* I	TQB72986.1, *CHS* VII	TQB70564.1, *CHS* II	TQB69157.1, *CHS* II	TQB73548.1, hypothetical protein	TQB73973.1, hypothetical protein	TQB73547.1, hypothetical protein		9
Monascus purpureus LQ-6		monascus_02563, *chs*2	monascus_02508, *chs*3	monascus_05161, *chs* 4	monascus_05162, *chs* 6	monascus_02870, *chs* activator	monascus_02765, *chs* 5	monascus_02400, *chs* G	monascus_04382, *chs* A			8
Monascus purpureus M183		g872, *chs* 2	g920, *chs* F	g3077, *chs*E	g3078, *chs*	g2747, *chs* 3	g5275, *chs* 3	g4739, *chs* B	g5640, *chs* A			8
